# ORFograph: search for novel insecticidal protein genes in genomic and metagenomic assembly graphs

**DOI:** 10.1186/s40168-021-01092-z

**Published:** 2021-06-28

**Authors:** Tatiana Dvorkina, Anton Bankevich, Alexei Sorokin, Fan Yang, Boahemaa Adu-Oppong, Ryan Williams, Keith Turner, Pavel A. Pevzner

**Affiliations:** 1 Center for Algorithmic Biotechnology, Saint Petersburg State University, Saint Petersburg, Russia; 2 Department of Computer Science and Engineering, University of California San Diego, San Diego, CA USA; 3 Université Paris-Saclay, INRAE, Micalis Institute, AgroParisTech, 78350 Jouy-en-Josas, France; 4 Data Science & Analytics, Bayer U.S. - Crop Science, Chesterfield, MO USA; 5 Ascus Biosciences, San Diego, CA USA; 6 Thermo Fisher Scientific, Carlsbad, CA USA

**Keywords:** Bioinformatics, Gene finding, Bacterial genomics, Metagenomics, Bioinsecticides

## Abstract

**Background:**

Since the prolonged use of insecticidal proteins has led to toxin resistance, it is important to search for novel insecticidal protein genes (IPGs) that are effective in controlling resistant insect populations. IPGs are usually encoded in the genomes of entomopathogenic bacteria, especially in large plasmids in strains of the ubiquitous soil bacteria, *Bacillus thuringiensis* (*Bt*)*.* Since there are often multiple similar IPGs encoded by such plasmids, their assemblies are typically fragmented and many IPGs are scattered through multiple contigs. As a result, existing gene prediction tools (that analyze individual contigs) typically predict partial rather than complete IPGs, making it difficult to conduct downstream IPG engineering efforts in agricultural genomics.

**Methods:**

Although it is difficult to assemble IPGs in a single contig, the structure of the genome assembly graph often provides clues on how to combine multiple contigs into segments encoding a single IPG.

**Results:**

We describe ORFograph, a pipeline for predicting IPGs in assembly graphs, benchmark it on (meta)genomic datasets, and discover nearly a hundred novel IPGs. This work shows that graph-aware gene prediction tools enable the discovery of greater diversity of IPGs from (meta)genomes.

**Conclusions:**

We demonstrated that analysis of the assembly graphs reveals novel candidate IPGs. ORFograph identified both already known genes “hidden” in assembly graphs and potential novel IPGs that evaded existing tools for IPG identification. As ORFograph is fast, one could imagine a pipeline that processes many (meta)genomic assembly graphs to identify even more novel IPGs for phenotypic testing than would previously be inaccessible by traditional gene-finding methods. While here we demonstrated the results of ORFograph only for IPGs, the proposed approach can be generalized to any class of genes.

**Video abstract**

**Supplementary Information:**

The online version contains supplementary material available at 10.1186/s40168-021-01092-z.

## Introduction

*Biopesticides* are important components of pest management programs that have been successful as an alternative to conventional chemical pesticides. These compounds, which are developed from the plant, animal, and bacterial proteins, do not leave harmful residues, are non-toxic to humans and the environment, and are more target-specific than conventional pesticides [51]. These advantages led to a worldwide proliferation of biopesticides and resulted in a multi-billion-dollar biopesticide market.

*Insecticidal proteins*, representing an important class of biopesticides, have been widely used in agriculture. Entomopathogenic bacteria, especially strains of the species *Bacillus thuringiensis* (*Bt*), produce *crystal* (*Cry*) and *cytolytic* (*Cyt*) insecticidal proteins and secreted *vegetative* insecticidal proteins (*VIPs*) that specifically target various insects, including insects from the orders Lepidoptera, Coleoptera, Hemiptera, and Diptera [51]. Insecticidal proteins are used to control pests of crop plants by mechanical methods, such as spraying to disperse microbial formulations containing various bacterial strains onto plant surfaces, and by using genetic transformation techniques to produce transgenic plants expressing insecticidal proteins. Indeed, the development of insecticidal transgenic crops has been transformative for agriculture. In 2017, 101 million hectares of cropland were devoted to their cultivation across the world and the adoption of specific transgenic crops has been associated with the reduction or elimination of broad-spectrum synthetic chemical insecticides in those environments [56].

Although insecticidal proteins from *B. thuringiensis* have become an important biopesticide against a wide range of insects, their prolonged use leads to rapidly developing *toxin resistance* [24]. Thus, it is important to search for novel insecticidal proteins that are effective in controlling resistant insect populations. Although the number of known Cry-encoding genes grew from just 14 30 years ago [30] to over 700 today, there is a constant need to identify new insecticidal protein genes (IPGs) to overcome insecticide resistance. Since *B. thuringiensis* is indigenous to many environments (its strains have been isolated worldwide from soil, insects, and leaves), genomic and metagenomic samples containing *B. thuringiensis* or other entomopathogenic bacterial strains provide many opportunities for finding novel IPGs [59]. However, the search for novel IPGs faces computational challenges that we describe below.

Initially, the Cry-encoding genes were searched for by PCR-based techniques using primers from their highly conserved regions [6, 10]. The basic PCR step was followed by variations such as E-PCR [35], PCR-RFLP [29], and PCR-SSCP [39]. Historically, these methods, which are all limited by the success/failure of the primer selection had only been applied to the discovery of the three-domain Cry genes [48].

Next-generation sequencing opened new possibilities for IPG discovery as novel Cry and Cyt genes in a newly sequenced genome can be found by similarity search against a database of known genes [52]. In particular, the similarity search based on *Hidden Markov Models* (*HMMs*) allows one to reveal more diverged Cry genes than those found using PCR-based methods. However, since Cry genes are rather variable, their HMMs typically represent only the main sequence domains rather than complete Cry genes. Ye et al. [67] and Zheng et al. [70] developed the BtToxin_scanner and BtToxin_Digger tools that use machine learning techniques to make the search for Cry genes more sensitive. BtToxin_scanner was applied for Cry gene identification in various studies [13, 19, 57].

However, all existing methods for IPG discovery are limited in their ability to reconstruct complete genes when their fragments scattered over multiple contigs. Since popular general-purpose gene prediction tools GeneMark [7], Prodigal [32], and Glimmer [20], as well as their metagenomic versions metaGeneMark [72], metaProdigal [33], and metaGlimmer [37], analyze individual contigs, they typically predict partial rather than complete IPGs, a bottleneck in the downstream IPG engineering efforts in agricultural genomics.

Development of a candidate IPG into a commercially viable toxin is a complex and time-consuming process that includes (i) prioritization of novel candidate IPGs for follow-up synthesis, (ii) synthesis and expression of selected IPGs for follow-up novel toxin production, and (iii) testing these novel toxins against various insects. ORFograph contributes to the first step of this pipeline by providing additional candidate IPGs whose parts are scattered over multiple contigs and thus were not available for a follow-up analysis in previous studies. This new stream of novel IPGs is important not only for agricultural genomics but also for biomedicine since some Cry proteins, such as parasporins, preferentially kill cancer cells [50].

ORFograph searches for novel IPGs in the *assembly graphs* (rather than individual contigs) that are generated by modern genome assemblers such as SPAdes [5] and metaSPAdes [49]. Given a read-set, SPAdes and metaSPAdes first construct the *de Bruijn graph* that consists of *nodes* (*k*-mers that appear frequently in reads) and *edges* connecting these nodes that are labeled by substrings from reads [17]. Since each error in a read creates a *bubble* in the de Bruijn graph (making this graph very complex), SPAdes and metaSPAdes error-correct reads and transform the de Bruijn graph into a simpler *assembly graph*. In the case of an “ideal” assembly graph, a genome is spelled by a path that visits all edges of the assembly graph.

Given a read-set, an assembly graph consists of *nodes* (*k*-mers that appear frequently in reads) and *edges* connecting these nodes that are labeled by substrings from the genome [17]. In the case of an “ideal” assembly graph, a genome is spelled by a path that visits all edges of the assembly graph.

**Fig. 1:**
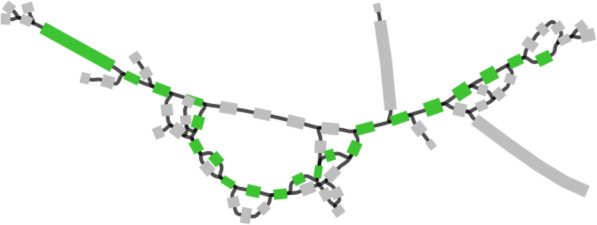
A subgraph of the SPAdes assembly graph of the SRR6238356 dataset reveals a large number of potential IPGs. Green edges represent a path that spells one out of ~3000 potential IPGs in this subgraph. All potential IPGs arising from this subgraph form four clusters and represent Cry1-like genes. The SPAdes assembly graph was constructed in the iterative mode with the default *k*-mer sizes (21, 33, 55). The subgraph was visualized using Bandage [65]

Figure 1 presents a small subgraph of the assembly graph of the SRR6238356 dataset constructed by SPAdes. The entire assembly graph consists of 1732 vertices and 1288 edges (654 of them are long edges with lengths exceeding 1000 bp). The green path in Fig. 1 represents one of three potential Cry1-like genes in this subgraph that has a length 3378 bp and traverses 21 edges. The existing gene prediction tools run on linear contigs and are not designed to predict genes on graphs. Therefore, if an IPG is scattered over multiple contigs, these tools can at best predict some fragments of this gene (losing information about the order of these fragments) rather than a complete gene, thus impairing any further IPG engineering efforts.

Figure 1 illustrates the importance of “threading” a known IPG (or an IPG domain represented by an HMM) through the assembly graph to discover novel IPGs. This *sequence-to-graph alignment problem* has been addressed for gene prediction in splicing graphs [25], for protein alignments in a graph describing all potential secondary structure predictions [68], and for gene prediction in metagenomic assembly graphs [, 31, 64, 66, 71]. However, since the existing sequence-to-graph alignment approaches lack the ability to align HMMs to metagenomic assembly graphs and do not take into account specific features of IPGs, they are not well suited for IPG discovery.

Here, we describe the ORFograph algorithm (the source code is publicly available at https://github.com/ablab/orf-search) and apply it to all publicly available read-sets representing the *Bacillus* genus as well as to an underexplored metagenomic datasets. ORFograph uses the SPAligner tool for graph-based sequence alignment [21] and the PathRacer tool for graph-based HMM alignment [62] to find novel IPGs (scattered over multiple contigs) that evaded detection by previous approaches. ORFograph identified nearly a hundred novel IPGs that evaded detection in all previous studies. Our work demonstrates that traditional “single contig” gene-finding approaches (both *ab initio* and similarity-based [46]) should be complemented by graph-based gene-prediction algorithms that use databases of proteins and protein domain models as additional evidence for finding genes in (meta)genomes. These graph-based algorithms can contribute to unmasking gene content and diversity found in (meta)genomes, especially for large and variable gene families. Although ORFograph is currently limited to insecticide toxins, our next goal is to extend it into a general tool for identifying arbitrary protein families in assembly graphs such as glycoside hydrolases and CAS proteins that are often scattered over multiple contigs.

## Results

### ORFograph pipeline

**Fig. 2:**
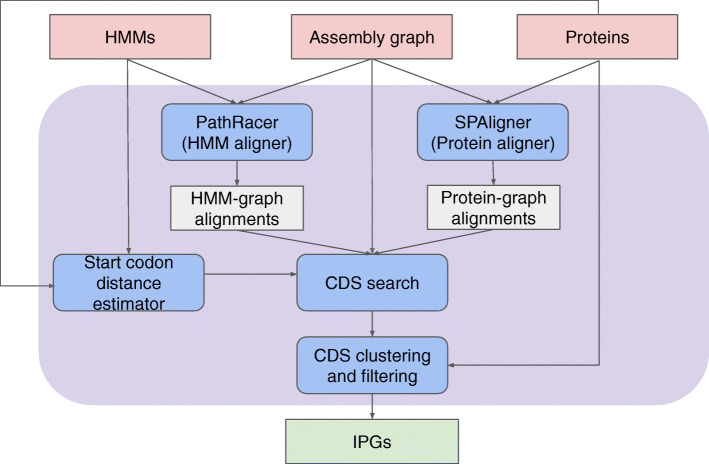
The ORFograph pipeline

After constructing the assembly graph, ORFograph searches for IPGs encoded in this graph. Below we describe the steps of the ORFograph pipeline (Fig. 2):

1. *Aligning known insecticide proteins/HMMs to the assembly graph*. ORFograph uses SPAligner [21] to align known insecticide proteins to the assembly graph and retains all alignments with a length exceeding 80% of the protein length. It also uses PathRacer [62] to align insecticide HMMs to the assembly graph and retains all alignments with e value below 10^−9^ and length exceeding 90% of the HMM length. These alignments reveal *anchor-paths* (partial ORFs) that have to be further extended into complete ORFs. An anchor-path either traverses a substring of an edge, or an entire edge, or multiple edges in the assembly graph.
2. *Start and stop codon search*. For each anchor-path, ORFograph finds all putative start and stop codons in the assembly graph by exploring all paths in this graph as described in the “Methods” section.
3. *Generation of complete coding sequences* (*CDSs*). Given a graph, we refer to the set of all paths between its vertices *v* and *w* (found by bounded exhaustive search limited to generating at most 1000 paths) as *Paths*(*v,w*). Given an anchor-path *AnchorPath* between vertices *start*(*AnchorPath*) and *end*(*AnchorPath*), a start codon ending at vertex *start-codon* and a stop-codon starting at vertex *stop_codon*, ORFograph generates path-sets *Paths*(*start-codon*,*start*(*AnchorPath*)) and *Paths*(*end*(*AnchorPath*)*,stop_codon*). For each pair of paths *PathFromStart* and *PathToStop* from the constructed path-sets, it further constructs the concatenate formed by the start codon, *PathFromStart*, *AnchorPath*, *PathToStop*, and stop codon. ORFograph considers only the concatenated paths with length below *max_restorable_length* threshold (default value 3000). This operation is repeated for each anchor-path and each pair of start/stop codons, followed by filtering of the concatenated paths as described in “Methods” section. Finally, each resulting path (in nucleotides) is translated into the corresponding amino acid sequence and duplicates are filtered out.
4. *IPG clustering and selecting representative IPGs*. In the case of complex assembly graphs, ORFograph may output dozens (and even hundreds) of very similar IPGs, thus complicating further analysis. ORFograph thus clusters the resulting potential IPGs and selects a *representative IPG* in each cluster (see “Methods” section). Since this paper focuses on difficult-to-find IPGs scattered over multiple contigs, easy-to-identify sequences that are found in a single contig and sequences that represent known genes can be filtered out from the main output.

### Datasets

We benchmarked ORFograph using the following (meta)genomic datasets publicly available at https://figshare.com/s/f20604a5333bbe4514c9:

#### Bti1 and Bti2 datasets

These two Illumina read-sets (accession numbers SRR8474067 and SRR8467560) correspond to *Bti* strains ATCC35646 and AM65-52 that are further referred to as Bti1 and Bti2, respectively [8]. ATCC35646 is a type strain of *Bti* often designated as ATCC35646. AM65-52 is the main component of the Vectobac®, a water-dispersible powder formulation of *Bti* for control of mosquito larvae. Both datasets were assembled with SPAdes (Table 1).

**Table 1:** Read and assembly statistics for Bti1, Bti2, and NYCS datasets

Dataset	#reads (millions)	#long edges in the assembly graph	Total assembly length (Mb)	N50 (kb)
Bti1	47	1268	6.4	98
Bti2	17	490	6.5	157
NYCS	5	2968	12.5	133

Statistics were generated with QUAST [27] and MetaQUAST [44] tools. The number of long edges (defined as edges longer than 1 kb) reflects the complexity of the assembly graph. The reads were trimmed and filtered using Trimmomatic-0.38 (with parameters: ILLUMINACLIP:adapters/TruSeq3-PE.fa:2:30:10 LEADING:3 TRAILING:3 SLIDINGWINDOW:4:15 MINLEN:70) and assembled with SPAdes-3.12 (Bti1 and Bti2) and metaSPAdes-3.12 (NYCS) with *k*-mer size 55.

#### All isolate IPG-containing bacteria in the NCBI database (B_ALL_ dataset)

To extract all *Bacillus* datasets from the NCBI database, we used the search query (((Bacillus[Organism]) AND Illumina) AND WGS[Strategy]) AND Paired[Layout]) that resulted in 2749 datasets. We used the Diamond tool [9] to align reads from each of the extracted datasets to known IPGs and detect datasets that contain Cry, Cyt, or Vip toxin genes. We say that an IPG is *covered* by a read-set if at least 50% of the IPG length is covered by reads alignments with identity exceeding 80%. A read-set was considered for further analysis if its reads covered at least one known IPG, resulting in 342 datasets. Afterward, we assembled each selected dataset using SPAdes and analyzed the resulting assemblies with ORFograph. ORFograph identified 72 datasets (among all 342 B_ALL_ datasets) that have IPGs alignments scattered over multiple contigs (Supplementary Table S2). Supplementary Figure S1 presents information about assemblies of these 72 datasets.

#### New York City subway metagenome (NYCS)

Afshinenkoo et al. [1] explored the microbial diversity of New York City subways by analyzing read-sets from multiple metagenomic samples (that we refer to as the NYCS dataset). Although this study did not specifically pursue the goal of finding IPGs, Parks et al. [53] identified a metagenome-assembled genome *B. thuringiensis* UBA3967 in some of these samples (Illumina read-set with accession number SRR1748627). Gillis et al. [26] analyzed the *B. thuringiensis* UBA3967 strain and noticed that, although it is very similar to the *Bti* genomes, it cannot be classified as a *Bti* strain since it lacks the *Bti* plasmids. Since all known Cry-encoding genes reside on plasmids, Gillis et al. [26] came to a conclusion that the reported *B. thuringiensis* UBA3967 assembly does not encode the entire set of Bti Cry toxins. We used ORFograph to search for potentially missed Cry and Cyt toxins in the metagenomic dataset from which the *B. thuringiensis* UBA3967 sequence was inferred.

#### Simulated datasets

In addition to real metagenomic datasets, we analyzed the simulated dataset generated by the Critical Assessment of Metagenome Interpretation (CAMI) consortium [63], complemented by simulated reads from various Bt strains that are enriched by the Cry genes. Supplementary Note “Benchmarking ORFograph on simulated datasets” benchmarks ORFograph on these datasets.

### Analyzing IPGs predicted by ORFograph

We used Bti1, Bti2, B_ALL_, and NYCS datasets to analyze ORFograph predictions. For each dataset, we launched SPAdes (Bti1, Bti2, and B_ALL_ datasets) or metaSPAdes (NYCS dataset) to construct the assembly graph and further launched the ORFograph pipeline applying SPAligner (using all known Cry, Cyt, and Vip toxins) and PathRacer (using all known HMMs derived from these toxins). Supplementary Table S1 provides information about ORFograph runtime and memory footprint.

Shikov et al. [61] recently developed the CryProcessor pipeline for IPG discovery by applying the PathRacer tool [62] to an assembly graph. However, since CryProcessor only searches for the three-domain Cry toxins, it cannot be used as a general pipeline for IPGs discovery from assembly graphs. It cannot be benchmarked against ORFograph since it only takes into account the sequences found by PathRacer rather than the paths that contain these sequences.

### ORFograph results for Bti1 and Bti2 genomes

Previous studies identified two genes encoding Cry proteins in Bti1 and seven genes encoding Cry proteins in Bti2 [8]. Both Bti1 and Bti2 have the pBtic100 plasmid carrying *cry60Aa* and *cry60Ba* genes. In addition, Bti2 has the pBtoxis plasmid with five Cry genes (*cry4Aa*, two *cry4Ba*, *cry10Aa*, *cry11Aa*) and three Cyt genes (*cyt1Aa*, *cyt2Ba*, *cyt1Ca*).

ORFograph identified 17 (23) clusters of putative IPGs in Bti1 (Bti2). We selected cluster representatives and ran BLAST [2] against the non-redundant protein database. Although most of these representatives have > 99% identity with thioredoxins, metallophosphoesterases, and disulfide reductases, ORFograph also identified *cry60Aa* and *cry60Ba* toxins in Bti1 that each resided in a single contig (and thus can be found without using ORFograph). In the case of Bti2, it identified *cry60Aa*, *cry60Ba*, five other known toxins (*cry11Aa*, *cry10Aa*, *cry4Aa*, and *cry4Ba*), and three known Cyt proteins (*cyt1Aa*, *cyt2Ba*, and *cyt1Ca)*.

**Fig. 3:**
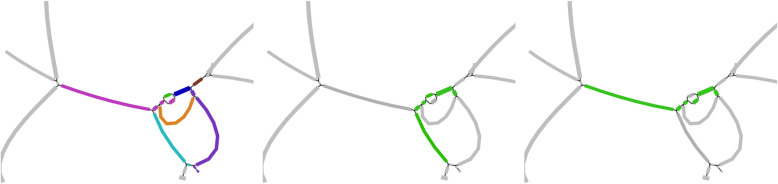
A subgraph of the Bti2 assembly graph aligned to the *cry4Aa* and *two cry4Ba* proteins. (Left) A subgraph of the Bti2 assembly graph with contigs represented as edges of the same color. (Middle) The *cry4Aa* gene path. (Right) The *cry4Ba* gene paths. Since one *cry4Ba* gene sequence is a substring of another, they belong to the same cluster. Subgraphs were visualized using the Bandage tool [65]

Proteins *cry11Aa*, *cry10Aa*, *cry60Aa*, and *cry60Ba* and all three Cyt proteins are located within single contigs and thus can be found without using ORFograph. However, *cry4Aa* and *cry4Ba* are scattered across several contigs and thus would evade identification by existing gene prediction tools (Fig. 3). Identification of these genes by ORFograph is particularly important since they contribute to the most valuable toxin activity against mosquitoes [58].

Gene-paths for two copies of *cry4Ba* coincide since one of them is a substring of another. The *cry4Aa* toxin was found at the ORFs generation step but was filtered out as its path conflicts with one of the contigs shown in dark magenta in Fig. 3. This filtering is based on the concept of “unique edges” that are currently defined based on a fixed threshold of 300 nt (see the “Methods” section), pointing to the challenge of parameter selection in ORFograph. Since the Bti2 dataset resulted in a high-quality assembly (N50 = 157kb), increasing this threshold for assemblies with high N50 (e.g., from 300 nt to 500 nt) would lead to identifying *cry4Aa*. In the future, we will modify ORFosearch to make this threshold variable rather than fixed.

As most Cry genes reside on plasmids, we also checked if the specialized plasmid assembler plasmidSPAdes [3] can resolve the *cry4* gene-paths in the Bti2 assembly graph. We launched plasmidSPAdes with default parameters on Bti2 reads and restored the same set of Cry genes as with the standard assembly approach.

Although plasmidSPAdes succeeded in this (relatively simple) case, our analysis revealed that it results in an only modest contribution to ORFograph identifications as compared to SPAdes. Supplementary Note “Benchmarking ORFograph against plasmidSPAdes and metaplasmidSPAdes” analyzes plasmidSPAdes and metaplasmidSPAdes [4] and demonstrates that these tools have limited benefits for IPG identification.

### ORFograph results for B_ALL_ datasets

ORFograph identified 72 datasets (among all 342 B_ALL_ datasets) that have IPGs alignments scattered over multiple contigs and selected 419 novel cluster representatives among them (Supplementary Table S2). One of B_ALL_ datasets (SRR6238356) contained a very large number of potential IPGs, most of which arise from traversing a highly complex area of the assembly graph (Fig. 1). While this dataset greatly increased the number of potential IPGs (increasing it from 2488 to over 5600), it had only 40 representatives, that were included in the follow-up analysis.

**Fig. 4:**
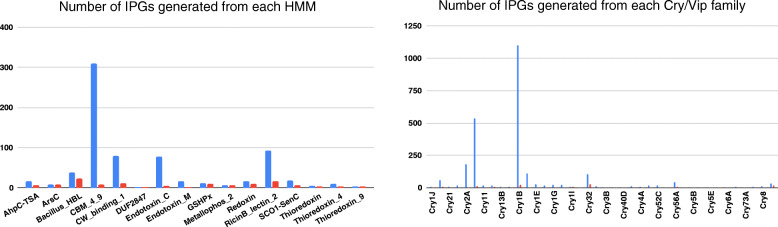
HMMs and Cry families that led to identifying potential IPGs in the B_ALL_ datasets. Histograms show the number of IPGs generated from alignments of HMMs (left) and genes from the Cry/Vip families (right). The blue columns show the number of novel putative IPGs that were generated from an alignment of a particular HMM (left) or Cry family (right), while the red columns show how many of these IPGs were selected as cluster representatives. The histogram on the right does not account for unclassified Cry1, Cry1A, and Cry1C families since they had a much larger number of sequences (536, 2382, and 1087, respectively)

Unclassified Cry1, Cry1A, and Cry1C are the most productive Cry families that served as seeds for identifying most IPGs (≅ 3522 ORFs and ≅ 122 cluster representatives in total). CW_binding_1 and Bacillus HBL are the most productive HMMs (Fig. 4).

#### Reliable and candidate IPGs

Since all gene prediction tools output some false positives, they face the challenge of assigning some measure of reliability to the predicted genes [28]. For example, short predicted genes are typically less reliable than long predicted genes. We thus analyzed the similarities between cluster representatives and known IPGs to classify the predicted IPGs into *reliable* (with similarity above a threshold) and *candidate* (with similarity below a threshold). We emphasize that candidate IPGs are not necessarily incorrect since they may represent particularly interesting cases of novel IPGs that have limited similarity with known IPGs.

**Fig. 5:**
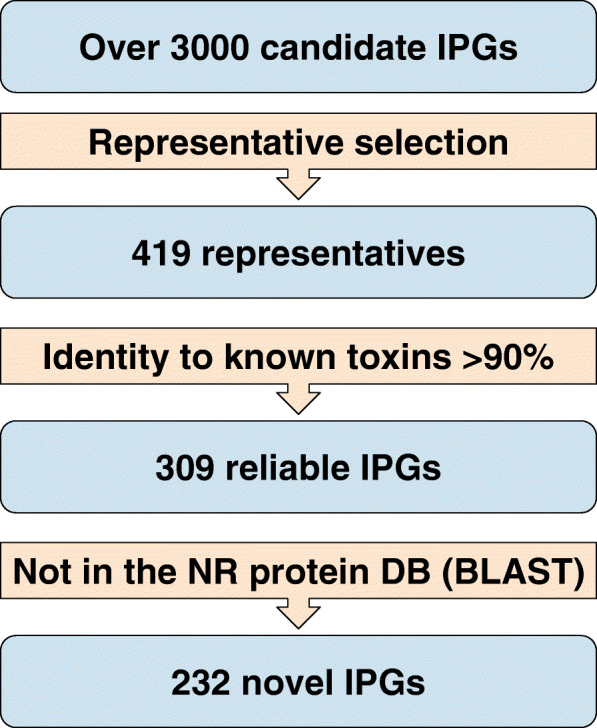
Outline of a step-by-step process for generating a shortlist of novel IPGs in the B_ALL_ datasets

Since the Bacterial Pesticidal Protein Resource Center [16] uses a rather stringent criteria for identifying reliable novel IPGs (at least 95% amino acid identity (AAI) to an existing IPG), we decided to use a less stringent criteria (at least 80% AAI to an existing IPG) to identify IPGs that significantly diverged from known IPGs. We classify a reliable IPGs as *novel* if it has AAI exceeding 90% (but less 100%) to a known IPG from the BLAST database. Three hundred nine out of 419 cluster representatives were classified as reliable and 232 of them were classified as novel (Fig. 5 and Supplementary Table S3).

**Fig. 6:**
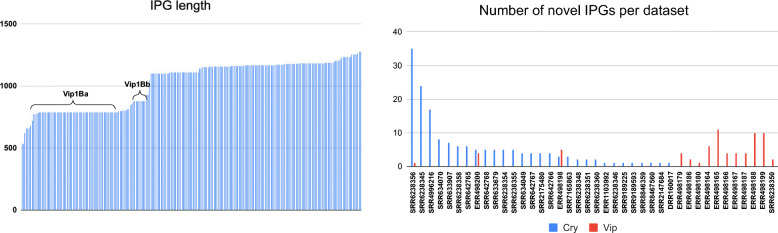
Statistics of 232 novel putative IPGs. (Left) The length distribution of 232 (164 Cry and 68 Vip) novel putative IPGs (in amino acids). (Right) The number of the novel putative IPGs generated per dataset. Only 40 datasets that contain 232 IPGs are shown

One hundred forty-five out of 232 novel IPGs have the length of 1000-1200 amino acids (Fig. 6). Sixty-seven candidate IPGs with length around 750-900 bp are similar to Vip1B, while other 20 candidate IPGs shorter than 1000 bp are similar to Cry2, Cry4, Cry5, Cry11, Cry13, Cry27, and Vip3 families. Most of 145 longer sequences have high identity with sequences from Cry1 family (Fig. 6, left) and only 8 of them are similar to Cry4, Cry5, and Cry9 genes.

Figure 6, right illustrates that just three datasets (SRR6238356, SRR6238345, and SRR4996216) contain 77 out of 232 reliable IPGs. The first two of these datasets were generated in Méric et al. [43] where the authors analyzed the transfer of Cry-rich plasmids between various species. Méric et al. [43] analyzed assemblies of 190 *B. cereus* group isolates, identified genes encoding Bt toxins (Cry, Cyt, Vip, and Sip) using BtToxin_scanner, and revealed that most Cry gene variants belong to Cry1Ia2, Cry2Aa9, Cry2Ab3, and Vip3A families. Our analysis of these datasets is consistent with the results in Méric et al. [43] with respect to IPGs found in single contigs. However, neither of 59 potential IPGs identified by ORFograph was identified by BtToxin_scanner (Table S4 in [43]). In addition, two potential IPGs identified by BtToxin_scanner represent fragments (rather than complete genes) of the reliable IPGs identified by ORFograph.

SRR4996216 dataset (with 17 identified IPGs) contains Illumina reads from *B. thuringiensis* serovar *aizawai* strain HD-133 assembled via the A5-miseq pipeline [14] in the original study [34]. Genome annotation was carried out using Prokka [60] and the NCBI’s Prokaryotic Genome Annotation Pipeline. The original study revealed Cry1Aa, Cry1Ab, Cry1Ca, Cry1Da, Cry1Ia, Cry2Ab, and Cry9Ea, which include many partial or apparently fragmented genes. In contrast, ORFograph found 16 full IPGs, including novel variants of Cry1Aa, Cry1b, Cry1Ca, and Cry1Da.

All Vip1B-like IPGs were obtained from datasets generated in [41]), where authors characterized the pathogen genes involved in coevolutionary adaptation in an animal host-pathogen interaction system of *Caenorhabditis elegans* and *Bacillus thuringiensis*.

Our analysis of the diverse B_ALL_ datasets demonstrates that ORFograph greatly extends the set of IPGs found in previous studies. Although our analysis is not exactly benchmarking (since we are identifying sequences not previously assembled), Supplementary Figure S2 present a comparison of putative novel IPGs to existing annotations in the IPG database (the labeled nodes on the tree show known IPGs and the others show putative novel IPGs), illustrating the value of ORFograph as a tool for novel IPG discovery.

### ORFograph results for the NYCS dataset

**Fig. 7:**
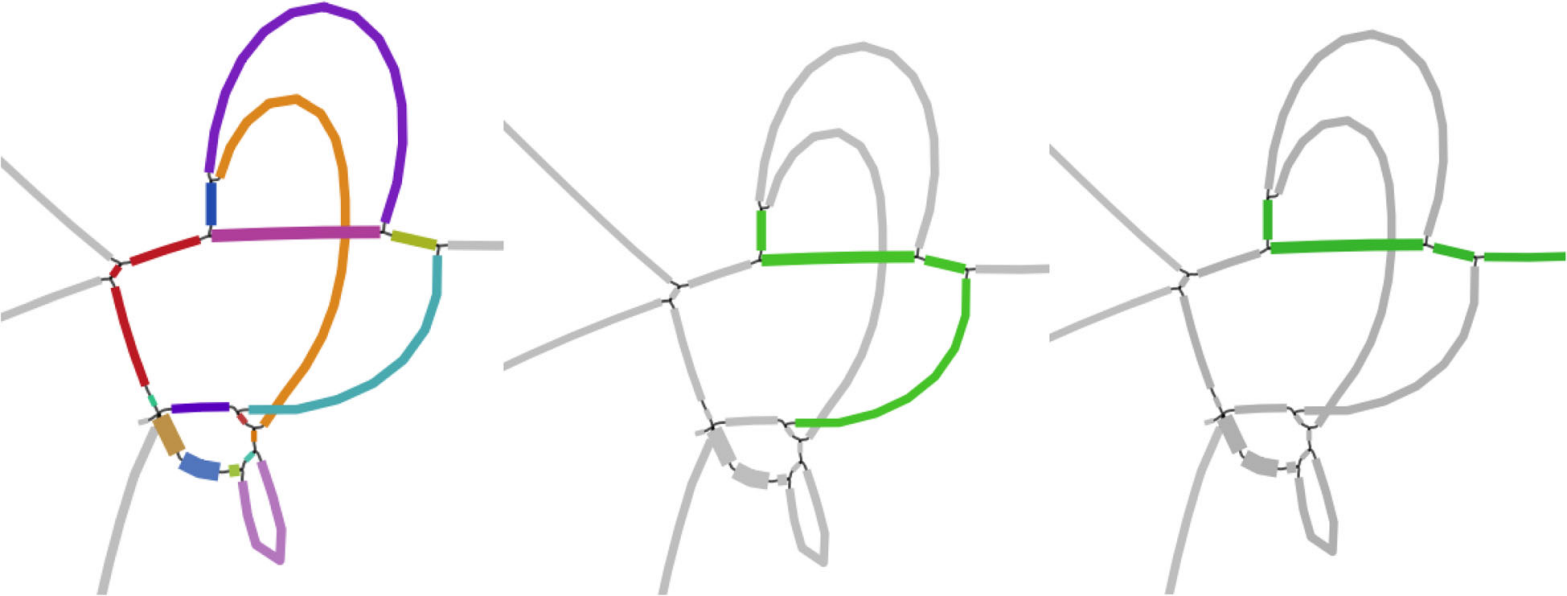
A subgraph of the NYCS assembly graph aligned to *cry4AA* and *cry4BA* (*cry4Ba*) proteins. (Left) A subgraph of the NYCS assembly graph with contigs represented as edges of the same color. (Middle) The *cry4AA* gene-path. (Right) The *cry4BA/cry4Ba* gene-path. Subgraphs were visualized using Bandage [65]

ORFograph found 48 clusters of putative IPGs in the NYCS metagenome assembly graph. We compared all cluster representative with the non-redundant protein database using BLAST. Similar to the analysis of genomic datasets, most cluster representatives have > 99% identity with thioredoxins, metallophosphoesterases, and disulfide reductases. However, ORFograph also identified *cry60Aa*, *cry60Ba*, *cry11Aa*, *cry10Aa*, *cry4Aa*, and two *cry4Ba* genes as well as three Cyt toxins. Similar to the Bti2 dataset, since *cry4* genes were scattered over several contigs (Fig. 7), they would evade identification without ORFograph. Since the NYCS dataset contains many Cry and Cyt genes that are similar to genes identified in the Bti2 dataset, we can conclude that UBA3967, also assembled from this dataset, represents mostly the chromosomal part of a viable *Bti* strain, but with only traces of plasmids that should also be there. We thus detected the corresponding toxins encoded by the plasmids, as it should be expected.

#### Previous analysis of B. thuringiensis UBA3967 strain

To analyze *B. thuringiensis* UBA3967 strain, Gillis et al. [26] compared all plasmids from the strain AM65-52 to all genome assemblies labeled as *B. thuringiensis* in NCBI (Table 2 in Gillis et al. [26] lists only those relevant to the *Bti* cluster). For the UBA3967 assembly, Gillis et al. [26] found only a rather short region of similarity with plasmids pBtic360, pBtoxis, and pBtic100. However, to conclude that a plasmid is present in the assembly, the similar region should typically cover almost the entire plasmid. Since it was not the case, Gillis et al. [26] concluded that no plasmid counterpart of those from AM65-52 exists in the UBA3967 strain. The found short similar regions may represent some insertions of transposable elements (IS-elements) or parts of Cry toxins existing in the assembly of the UBA3967 strain. Since all known environmental Bti isolates contain most of these plasmids, Gillis et al. [26] concluded that the UBA3967 assembly does not correspond to the entire genome (i.e., chromosome and plasmids) of a *Bti* isolate.

## Methods

### Constructing database of known IPGs and their Hidden Markov Models (HMMs)

**Fig. 8:**
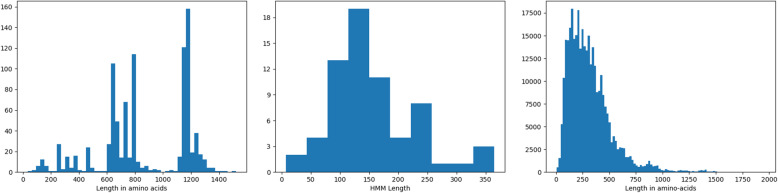
Histograms of the lengths of 941 known Cry and Vip insecticide proteins (left), 66 HMMs representing IPGs (middle), and all bacterial genes from the Uniprot database (right). 84% of the Cry and Vip genes have a length exceeding 600 amino acids (left) while only 7% of all genes have a length exceeding 600 amino acids (right)

Accession numbers of known Cry and Vip toxins were taken from the Bt nomenclature list (Crickmore et al. 2018), and sequences were downloaded from the NCBI database, forming the *IPG database*. This procedure resulted in extracting 941 protein sequences (811 Cry and Cyt toxins as well as 130 Vip toxins) with the average length of each protein approximately 800 amino acids (Fig. 8, left).

In addition to forming the IPG database, we selected 66 publicly available HMMs commonly found in IPGs and downloaded them from the Pfam database [45]. The number of hidden states in the selected HMMs (referred to as *HMM length*) varies from 25 to 350 states (Fig. 8, middle).

### Gene discovery in the assembly graphs

ORFograph first attempts to find *open reading frames* (*ORFs*) containing parts of Cry and Vip genes scattered over multiple contigs. Since Bt genomes typically contain multiple similar Cry and Vip genes, these genes are often fragmented in genomic assemblies. Since the vast majority of Cry and Vip genes are longer than typical bacterial genes (Fig. 8, right), they are more susceptible to fragmentation than other genes. This fragmentation further amplifies in assemblies of metagenomic samples that contain multiple Bt strains.

ORFograph attempts to find all paths in the assembly graph that represent Cry/Vip-encoding ORFs. It first constructs anchor-paths by performing the HMM-to-graph [62] or sequence-to-graph [21] alignments to detect partial ORFs and further extends each anchor-path into a complete ORF. Since HMMs for most Cry/Vip genes represent a short domain rather than a full gene sequence, ORFograph often finds a large number of short alignments, with each such alignment-path revealing only a part of the potential gene rather than a complete ORF. ORFograph removes such short alignment-paths from further consideration if they represent subpaths of longer (and thus more informative) alignment-paths resulting from longer gene segments. Specifically, if an anchor-path is a subpath of another anchor-path with the same frameshift, this anchor-path is filtered out. Afterward, ORFograph extends each remaining anchor-path to find the complete ORF containing it.

Since insecticide toxins share some protein domains with thioredoxins, metallophosphoesterases, and disulfide reductases (originating from alignments of thioredoxin, metallophos, and AhpC-TSA HMMs, respectively), many HMM alignments found by ORFograph arise from these three protein families rather than IPGs. To filter out these three protein families from the ORFograph output, we compare each putative IPG found by ORFograph against the database of known insecticidal toxins using BLAST and only retain IPGs that are similar to a known insecticide toxin (with percent identity exceeding a threshold *Identity* = 80%).

### Search for start and stop codons in IPGs

ORFograph uses HMMs and known IPGs to find the highest-scoring anchor-paths that correspond to a partial Cry or Vip gene sequence. However, it is unclear how to extend these partial sequences into a *complete coding region* of a gene (referred to as *CDS*) since the choice of the start and stop codon for a given anchor-path is often ambiguous. In addition, the selected start and stop codons are often connected with the anchor-path by multiple paths. ORFograph finds all possible start/stop codons that can be reached from the leftmost/rightmost position of the anchor-path.

For each anchor-path, ORFograph finds all putative start and stop codons in the assembly graph by exploring all paths in this graph using the *Breadth-First-Search* [18]. During this search, it assigns a *frameshift string* (of length 1, 2, or 3) that specifies the part of the last codon traversed on the way to this node. A vertex is classified as *terminal* if its frameshift string represents a stop codon. The Breadth-First-Search identifies all terminal vertices and stops further graph exploration in these vertices. Information about sequences with start codons that are positioned after a stop codon or the Shine-Dalgarno sequence in the graph is reflected in the CDS file.

### CDS generation

After identifying the set of start and stop codons for each partial alignment, ORFograph explores CDSs corresponding to these alignments. A CDS given by the partial alignment corresponds to a path between a pair of start and stop codons that passes through the partial alignment and represents a putative IPG. For each pair of start and stop codons, the path is divided into prefix (an unknown path from the start codon to the leftmost position of the alignment), middle (the known partial alignment), and suffix (an unknown path from the rightmost position of the alignment to the stop codon).

ORFograph performs an exhaustive search to generate all potential prefixes and suffixes separately and concatenates them with the middle part to construct a set of full paths that represent potential IPGs. To speed up CDS generation, ORFograph pre-calculates a set of edges that can be traversed on the way from potential start codons to the beginning of the anchor-path (and from the end of the anchor-path to the potential stop codons) using the *Depth-First Search* [18]. ORFograph only uses these edges (rather than all edges of the graph) to generate prefixes and suffixes and further concatenates each prefix, the anchor-path, and each suffix to generate the putative IPG. Since the number of putative full-length genes is typically large, ORFograph filters and ranks the constructed putative IPGs as described below.

### Analyzing HMM positions within IPGs

We aligned all HMMs to the IPG database using the HMMer tool [22] and constructed the distribution of their starting positions inside the IPGs. ORFograph uses this distribution to find the most likely distance from the start codon to the position of the HMMs within an IPG. We aligned each HMM to all known IPGs and generated a histogram of all distances from the gene start to the start of each full HMM alignment. As Supplementary Figure S3 illustrates, this distance is very conserved for some HMMs (e.g., PA14) and less conserved for other HMMs (e.g., Aegerolysin). We thus defined the *likelihood* of a given distance *d* as the fraction of distances in the interval [*d-binSize, d+binSize*] in the histogram (the default value *binSize=*150). Using the computed likelihoods, ORFograph derives a set of the most likely distances from the start codon for each HMM alignment in the assembly graph and uses these distances to find the most likely prefixes as the prefixes with likelihoods exceeding the default threshold (a similar procedure is used to find the most likely suffixes).

### Filtering putative IPGs that conflict with contigs

ORFograph compares each putative IPGs with the set of contigs output by SPAdes/metaSPAdes and filters out IPGs that “contradict” to the contig-set as described below.

**Fig. 9:**
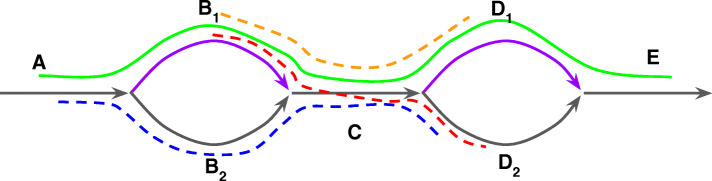
Examples of conflicting and non-conflicting gene paths in an assembly graph. Purple edges B_1_ and D_1_ are classified as unique but black edges B_2_ and D_2_ are classified as non-unique since they are shorter than the *minLength* threshold. Edges A, C, and E are classified as non-unique because their indegrees and/or outdegrees prevent their classification as unique edges. The green path conflicts with the red path but does not conflict with the blue and yellow paths. Purple edges represent unique edges of the green contig-path

While any genome segment is expected to be represented as a path (*correct path*) in the assembly graph, many paths do not correspond to genome fragments (*incorrect paths*). Figure 9 shows a fragment of an assembly graph that can be traversed in four different ways: AB_1_CD_1_E, AB_1_CD_2_E, AB_2_CD_1_E, and AB_2_CD_2_E. Each of these paths may be either correct or incorrect. Since alternative sequences in *bulges* (B_1_/B_2_ and D_1_/D_2_) are typically similar, it is likely that if one of them is chosen as an anchor-path, then another will be chosen as well. This effect can exponentially amplify the number of reported paths. Finding the correct path among many incorrect paths is not unlike the *repeat resolution* problem in genome assembly [5]. State-of-the-art genome assemblers solve this problem using a variety of additional information (e.g., paired-end reads) for finding correct paths (*contig*) in the assembly graph [54]. ORFograph takes advantage of contigs output by SPAdes/metaSPAdes and uses them to filter out incorrect IPGs as described below.

Two paths in the assembly graph are called *compatible* if they overlap (suffix of one of them coincides with a prefix of another) or if one of them is contained within another. ORFograph relies on *unique* edges in the assembly graph, i.e., the edges that are only passed once by the path representing the genome (*genome path*). If two correct paths both contain the same unique edge then these paths are compatible. Thus, any putative IPG that contains the same unique edge as a contig path and is not compatible with it can be filtered out as incorrect. In this case, we say that a putative IPG and a contig *contradict* each other.

Since the identification of unique edges in the assembly graph is a non-trivial problem, we use a simple (albeit error-prone) heuristic for finding them. An edge is considered unique if its length exceeds a threshold *minLength* (the default value 300 nucleotides), its start node has an outdegree larger than 1, and its end node has an indegree larger than 1. Figure 9 illustrates an example with a putative (red) IPG B_1_CD_2_ that contradicts the green contig AB_1_C and thus is filtered out. Although the putative (blue) IPG AB_2_CD_2_ shares an edge with the green contig, it does not contradict this contig because the shared edge is non-unique.

### IPG clustering

Even after applying the described filters, many putative IPGs may still remain, making it difficult to determine which of them are correct. ORFograph organizes putative IPGs into clusters using the single linkage clustering. Two sequences are clustered together if one of them is a substring of another or their percent identity exceeds a threshold *PI* (the default value *PI* = 90%). We further consider all edges of the assembly graph that contribute to IPGs in a given cluster and classify long (exceeding 1 kb) edges in the IPGs from a given cluster as *long cluster edges*.

### Selecting representative IPGs

In some cases, the set of putative IPGs remains large even after the filtering step. Since many of them may represent erroneous variants of the correct IPGs and since reporting all of them may be counter-productive for the downstream experimental analysis, ORFograph selects the most reliable putative IPGs as the best candidates for a further experimental analysis. Since long cluster edges (in difference from the potentially spurious short cluster edges) likely belong to some IPGs, we require that each long cluster edge belongs to at least one representative IPGs.

ORFograph selects a small set of the reliable potential IPGs in each cluster (*representative IPGs*) in such a way that paths spelled out by representative IPGs include each long cluster edge. For each path, we consider a triplet (*start codon distance likelihood*, *coverage by reads*, *path length*) and classify a path *P* as more reliable than a path *P’* from the same cluster if its triplet is lexicographically larger.

To select representative IPGs, we consider all potential IPGs in each cluster in a lexicographically non-decreasing order of their triplets. ORFograph scans the resulting ordered list and classifies an IPG in this list as representative if it contains a long edge that was not present in the previously considered IPGs from this list.

## Discussion

Although the recently developed SPAligner [21] and PathRacer [62] tools offer a possibility to search for any protein family in assembly graphs, we have decided to limit the scope of this project to insecticide toxins as each protein family has specifics that have to be taken into account during the tool development. Our next goal is to extend ORFograph into a general tool for identifying arbitrary protein families in assembly graphs such as glycoside hydrolases [36] and CAS proteins [40]. Existing tools for identifying protein families, such as GeneHunt [47], CasFinder [12], and HMMCAS [11] are limited to single contigs, thus missing proteins whose parts are scattered over multiple contigs. biosyntheticSPAdes [42] is the first tool from the SPAdes toolkit aimed at gene finding in assembly graphs. However, since biosyntheticSPAdes has a rather narrow focus on non-ribosomal peptide synthetases (NRPSs), it is not clear how to extend it to an arbitrary protein family.

ORFograph, currently limited to IPG finding, represents the first software from the SPAdes toolkit that can be easily extended into a more general tool for identifying an arbitrary protein family based on a set of family-specific HMMs and a set of approaches generalizing various metrics described in this paper. Development of such a general tool will enable mining breadth of genes from meta(genomes) for a variety of biotechnological uses, such as the discovery of novel antibiotic biosynthesis clusters, gene editing enzymes, or metabolic pathways for industrial biosynthetic use.

Although this paper analyzes IPGs predicted by ORFograph in the SPAdes assemblies, ORFograph can be applied to assembly graphs constructed by any genomic (e.g., Velvet [69]) or metagenomic (e.g., Megahit [38]) assembler.

It is important to note that *B. thuringiensis* is a member of the Bacillus cereus group and is closely related to the pathogenic *B. anthracis* and *B. cereus* strains. Since ORFograph facilitates high-throughput discovery of novel IPGs, it is important to verify that the newly discovered insecticide toxins do not harm humans.

## Conclusions

We demonstrated that analysis of the assembly graphs reveals novel candidate IPGs. ORFograph identified both already known genes “hidden” in assembly graphs and potential novel IPGs that evaded existing tools for IPG identification. As ORFograph is fast, one could imagine a pipeline that processes many (meta)genomic assembly graphs to identify even more novel IPGs for phenotypic testing than would previously be inaccessible by traditional gene-finding methods.

## Supplementary Information

**Additional file 1: Supplementary Table S1.** Information about ORFograph runtime and memory usage. ORFograph was run in 16 threads. **Supplementary Table S2.** ORFograph results for all 72 B_ALL_datasets with novel candidate IPGs. The “#novel candidate IPGs” column shows the number of ORFs that differ from known IPGs from the Bt nomenclature database. Datasets that produced reliable novel IPGs are marked as bold. **Supplementary Figure S1.** Assembly statistics for 72 datasets with potential novel IPGs. Each bar represents a measurement for one of 72 datasets. (Upper left) Number of reads per dataset (in millions); (Upper right) Number of long edges (exceeding 1 kb in length) in the assembly graph (in thousands). Number of ultralong edges (exceeding 5 kb in length) is shown in blue; (Bottom left) N50 of all contigs (in kb); (Bottom right) Total assembly length of long contigs (in megabases). Total assembly length of ultralong contigs is shown in blue. **Supplementary Figure S2.** Diversity of the reliable Cry1 sequences identified from the B_ALL_ dataset. Putative Cry1 novel IPGs identified by ORFograph were aligned with sequences from the Bt nomenclature list (Crickmore et al., 2018) using Muscle v3.8.31 [23], and a maximum likelihood phylogeny was constructed with FastTree v2.1.10 [55]. **Supplementary Figure S3.** Histograms of locations of matches of HMMs along the IPG sequences (only 14 out of 66 HMM have reliable matches). Orange (blue) columns show the distribution of distance from the gene starts (ends) to the location of the matches of HMMs. **Supplementary Note.** Benchmarking ORFograph on simulated datasets. **Supplementary Table S3.** Information about seven simulated datasets enriched with Cry genes. **Supplementary Table S4.** Information about the percent identity between six analyzed Cry proteins. Each cell shows the percent identity for between cry1aa1, cry1ab1, cry1ac1, cry1ca1, cry2aa1, and cry2ab1. **Supplementary Table S5.** Benchmarking ORFograph on five simulated datasets of varying complexity. **Supplementary Figure S4.** Most reliable IPGs (paths) identified by ORFograph in the Cry1Aa1+Cry1Ab1 dataset. Grey edges represent the subgraph containing all paths, while blue edges represent paths of four potential IPGs identified by ORFograph. In this subgraph, each edge represents a separate contig, illustrating that ORFograph faced the challenge of filtering spurious paths in the absence of the contig-related information from SPAdes. The shortest edge in the shown subgraph has length 348 bp. **Supplementary Figure S5.** The subgraph (grey edges) and the path (blue edges) of the Cry1-like IPG generated by ORFograph for the AllCry dataset. **Supplementary Note.** Benchmarking ORFograph against plasmidSPAdes and metaplasmidSPAdes.

## Data Availability

The datasets generated and/or analyzed during the current study are available at the figshare repository https://figshare.com/s/f20604a5333bbe4514c9
