# Supplementary material for: ORFograph: search for novel insecticidal protein genes in genomic and metagenomic assembly graphs
Source: Microbiome. 2021 Jun 28;9:149. doi: 10.1186/s40168-021-01092-z (PMC8240309; doi:10.1186/s40168-021-01092-z)
Supplement: Supplementary file 2 — Additional file 1: Supplementary Table S1. Information about ORFograph runtime and memory usage. ORFograph was run in 16 threads. Supplementary Table S2. ORFograph results for all 72 BALLdatasets with novel candidate IPGs. The “#novel candidate IPGs” column shows the number of ORFs that differ from known IPGs from the Bt nomenclature database. Datasets that produced reliable novel IPGs are marked as bold. Supplementary Figure S1. Assembly statistics for 72 datasets with potential novel IPGs. Each bar represents a measurement for one of 72 datasets. (Upper left) Number of reads per dataset (in millions); (Upper right) Number of long edges (exceeding 1 kb in length) in the assembly graph (in thousands). Number of ultralong edges (exceeding 5 kb in length) is shown in blue; (Bottom left) N50 of all contigs (in kb); (Bottom right) Total assembly length of long contigs (in megabases). Total assembly length of ultralong contigs is shown in blue. Supplementary Figure S2. Diversity of the reliable Cry1 sequences identified from the BALL dataset. Putative Cry1 novel IPGs identified by ORFograph were aligned with sequences from the Bt nomenclature list (Crickmore et al., 2018) using Muscle v3.8.31 [23], and a maximum likelihood phylogeny was constructed with FastTree v2.1.10 [55]. Supplementary Figure S3. Histograms of locations of matches of HMMs along the IPG sequences (only 14 out of 66 HMM have reliable matches). Orange (blue) columns show the distribution of distance from the gene starts (ends) to the location of the matches of HMMs. Supplementary Note. Benchmarking ORFograph on simulated datasets. Supplementary Table S3. Information about seven simulated datasets enriched with Cry genes. Supplementary Table S4. Information about the percent identity between six analyzed Cry proteins. Each cell shows the percent identity for between cry1aa1, cry1ab1, cry1ac1, cry1ca1, cry2aa1, and cry2ab1. Supplementary Table S5. Benchmarking ORFograph on five simulated datasets of varyi [file 40168_2021_1092_MOESM2_ESM.docx]

### **Supplementary Tables**

| **Dataset** | **Wall-clock time (hh:mm)** | **Peak RAM usage (Gb)** |
| --- | --- | --- |
| Bti1 | 00:03 | 0.3 |
| Bti2 | 00:13 | 1.6 |
| NYCS | 00:20 | 1.6 |

**Supplementary Table S1. Information about ORFograph runtime and memory usage.** ORFograph was run in 16 threads.

| # | Dataset | #identified IPGs | #IPGs scattered over multiple contigs | #novel candidate IPGs | #cluster representatives |
| --- | --- | --- | --- | --- | --- |
| 1 | **DRR160017** | 49 | 13 | 6 | 3 |
| 2 | DRR174869 | 218 | 111 | 45 | 10 |
| 3 | **ERR1103992** | 94 | 27 | 14 | 4 |
| 4 | ERR1104018 | 90 | 27 | 14 | 4 |
| 5 | ERR2221302 | 112 | 18 | 13 | 2 |
| 6 | **ERR498164** | 66 | 30 | 15 | 6 |
| 7 | **ERR498165** | 79 | 21 | 16 | 11 |
| 8 | **ERR498166** | 103 | 13 | 8 | 4 |
| 9 | **ERR498167** | 72 | 5 | 5 | 4 |
| 10 | ERR498173 | 43 | 18 | 12 | 4 |
| 11 | ERR498177 | 76 | 17 | 12 | 4 |
| 12 | **ERR498179** | 135 | 55 | 26 | 11 |
| 13 | **ERR498180** | 66 | 5 | 3 | 1 |
| 14 | ERR498182 | 96 | 47 | 31 | 12 |
| 15 | ERR498183 | 61 | 31 | 20 | 10 |
| 16 | ERR498184 | 60 | 30 | 19 | 9 |
| 17 | **ERR498186** | 49 | 8 | 4 | 2 |
| 18 | **ERR498187** | 52 | 8 | 5 | 4 |
| 19 | **ERR498188** | 78 | 19 | 17 | 10 |
| 20 | **ERR498198** | 273 | 102 | 41 | 12 |
| 21 | **ERR498199** | 248 | 78 | 45 | 10 |
| 22 | **ERR498200** | 755 | 644 | 359 | 20 |
| 23 | **SRR2147684** | 61 | 24 | 16 | 3 |
| 24 | **SRR2175480** | 207 | 124 | 62 | 4 |
| 25 | SRR3226043 | 61 | 12 | 8 | 2 |
| 26 | SRR392535 | 122 | 40 | 20 | 3 |
| 27 | SRR392536 | 120 | 40 | 20 | 3 |
| 28 | SRR392538 | 125 | 40 | 20 | 3 |
| 29 | SRR392546 | 86 | 4 | 2 | 2 |
| 30 | SRR392587 | 326 | 266 | 110 | 11 |
| 31 | SRR392676 | 148 | 54 | 24 | 2 |
| 32 | SRR398149 | 108 | 41 | 32 | 4 |
| 33 | SRR398165 | 70 | 25 | 20 | 4 |
| 34 | SRR4064643 | 103 | 22 | 17 | 3 |
| 35 | SRR4996210 | 113 | 18 | 13 | 2 |
| 36 | **SRR4996216** | 224 | 125 | 111 | 18 |
| 37 | SRR5121303 | 160 | 47 | 12 | 2 |
| 38 | SRR6012638 | 80 | 1 | 1 | 1 |
| 39 | SRR6012647 | 64 | 1 | 1 | 1 |
| 40 | SRR6012649 | 77 | 3 | 2 | 2 |
| 41 | SRR6012916 | 57 | 2 | 1 | 1 |
| 42 | SRR6019532 | 67 | 2 | 2 | 1 |
| 43 | **SRR6238345** | 838 | 595 | 459 | 39 |
| 44 | **SRR6238346** | 74 | 14 | 4 | 2 |
| 45 | **SRR6238348** | 89 | 27 | 14 | 5 |
| 46 | **SRR6238350** | 85 | 5 | 5 | 2 |
| 47 | **SRR6238351** | 133 | 52 | 20 | 2 |
| 48 | **SRR6238354** | 171 | 69 | 40 | 6 |
| 49 | **SRR6238355** | 133 | 71 | 42 | 8 |
| 50 | **SRR6238356** | 4303 | 3641 | 3168 | 40 |
| 51 | **SRR6238358** | 89 | 21 | 16 | 9 |
| 52 | **SRR6238360** | 77 | 21 | 12 | 3 |
| 53 | SRR6238362 | 235 | 98 | 57 | 10 |
| 54 | **SRR633679** | 208 | 126 | 63 | 5 |
| 55 | **SRR633907** | 214 | 128 | 66 | 7 |
| 56 | **SRR634049** | 192 | 124 | 62 | 4 |
| 57 | **SRR634070** | 202 | 130 | 67 | 8 |
| 58 | **SRR642765** | 244 | 126 | 64 | 6 |
| 59 | **SRR642766** | 206 | 124 | 62 | 4 |
| 60 | **SRR642767** | 267 | 164 | 88 | 4 |
| 61 | **SRR642768** | 209 | 124 | 61 | 5 |
| 62 | SRR642771 | 73 | 1 | 1 | 1 |
| 63 | **SRR7165863** | 129 | 43 | 23 | 6 |
| 64 | **SRR8467560** | 105 | 46 | 25 | 6 |
| 65 | SRR8846351 | 34 | 3 | 2 | 1 |
| 66 | SRR8846358 | 68 | 2 | 1 | 1 |
| 67 | **SRR8846359** | 26 | 7 | 3 | 1 |
| 68 | **SRR8846360** | 46 | 2 | 1 | 1 |
| 69 | SRR8846367 | 46 | 2 | 2 | 1 |
| 70 | SRR8846368 | 48 | 3 | 2 | 1 |
| 71 | **SRR9189225** | 56 | 1 | 1 | 1 |
| 72 | **SRR9189593** | 42 | 1 | 1 | 1 |

**Supplementary Table S2. ORFograph results for all 72 B_ALL_datasets with novel candidate IPGs.** The “#novel candidate IPGs” column shows the number of ORFs that differ from known IPGs from the Bt nomenclature database. Datasets that produced reliable novel IPGs are marked as bold.

**Supplementary Figures**


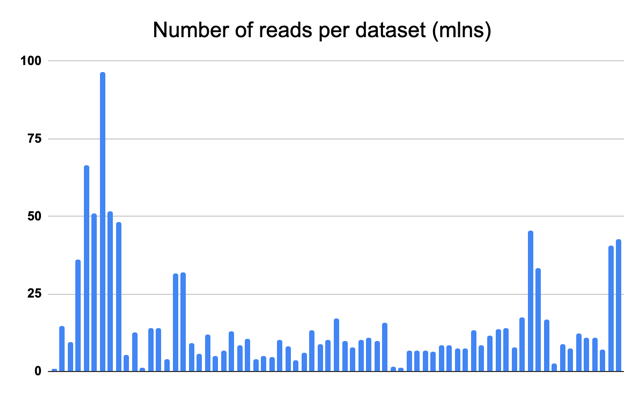

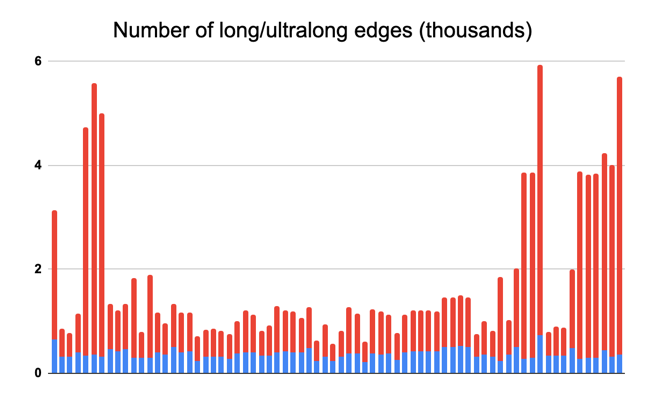


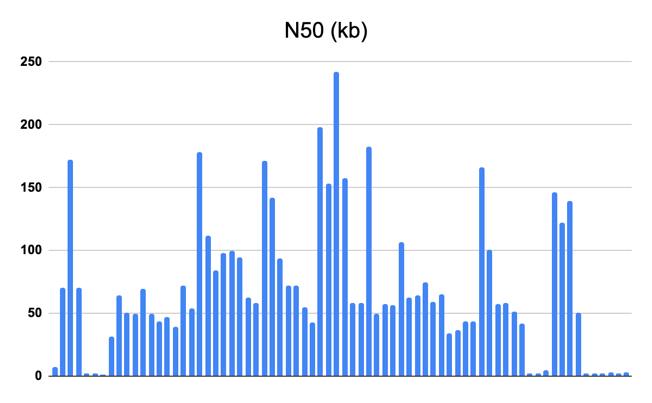

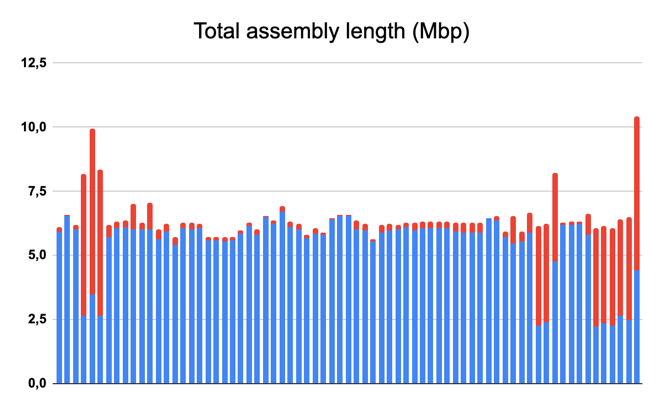


**Supplementary Figure S1. Assembly statistics for 72 datasets with potential novel IPGs.** Each bar represents a measurement for one of 72 datasets. (Upper left) Number of reads per dataset (in millions); (Upper right) Number of long edges (exceeding 1 kb in length) in the assembly graph (in thousands). Number of ultralong edges (exceeding 5 kb in length) is shown in blue; (Bottom left) N50 of all contigs (in kb); (Bottom right) Total assembly length of long contigs (in megabases). Total assembly length of ultralong contigs is shown in blue.


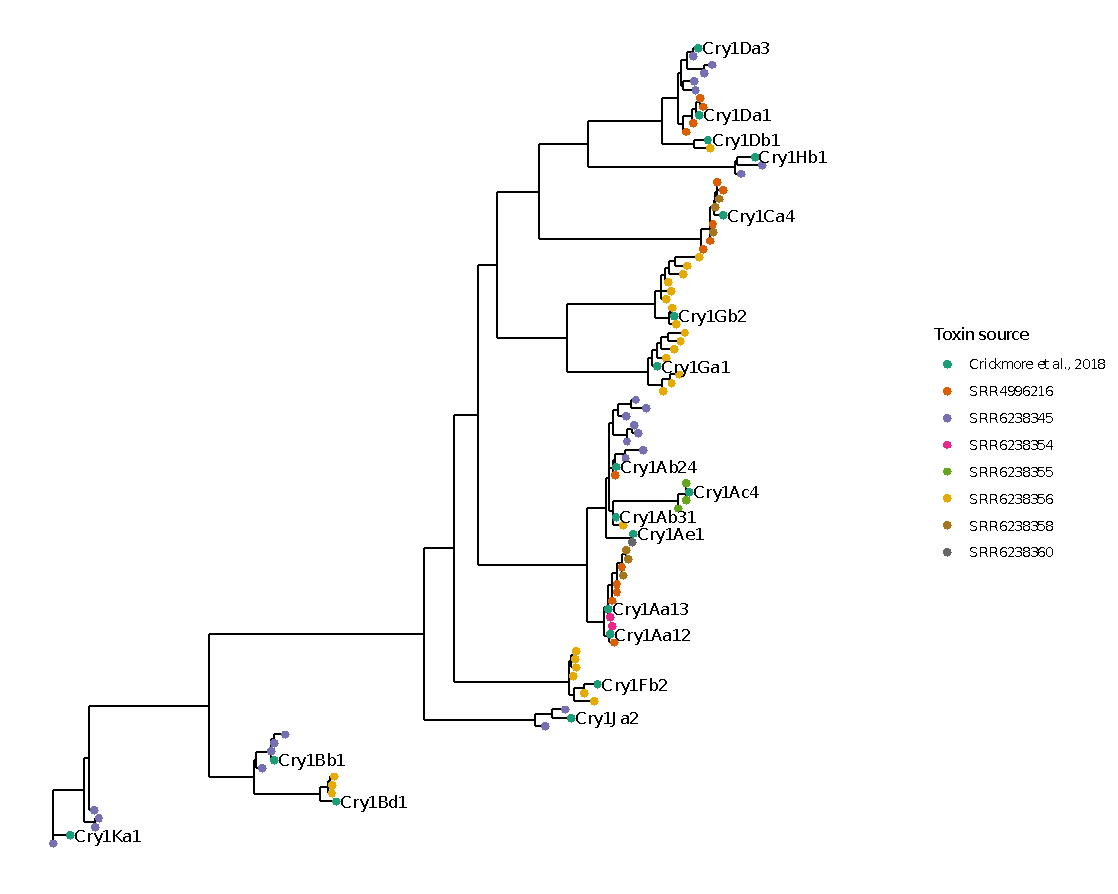
**Supplementary Figure S2. Diversity of the reliable Cry1 sequences identified from the B_ALL_ dataset.** Putative Cry1 novel IPGs identified by ORFograph were aligned with sequences from the Bt nomenclature list [16] using Muscle v3.8.31 (Edgar, 2014), and a maximum likelihood phylogeny was constructed with FastTree v2.1.10 (Price et al., 2010).


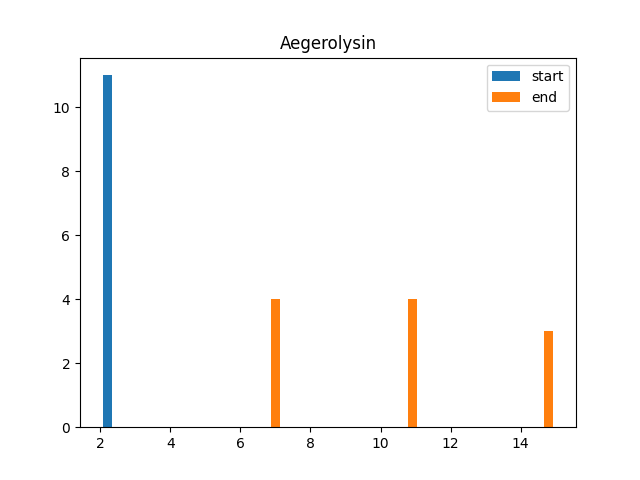

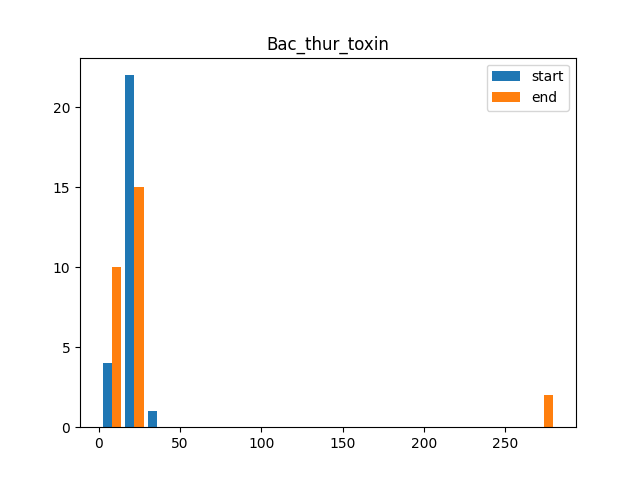


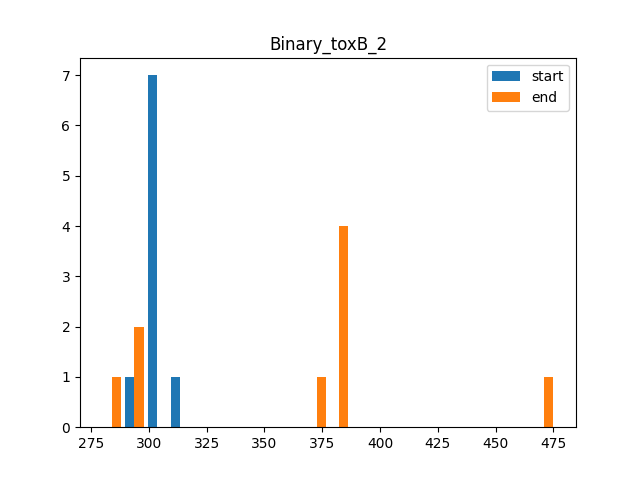

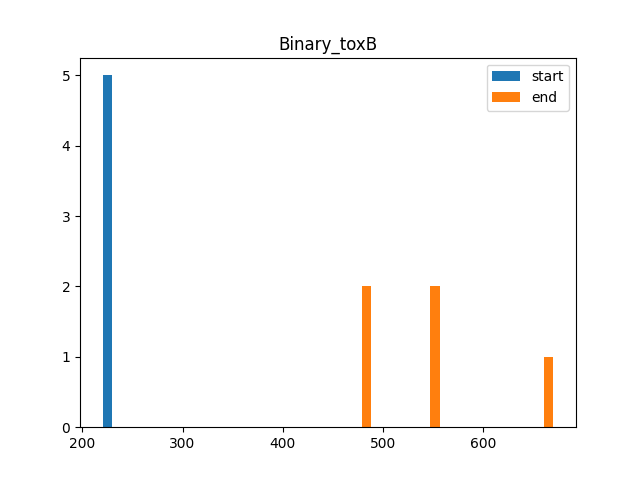


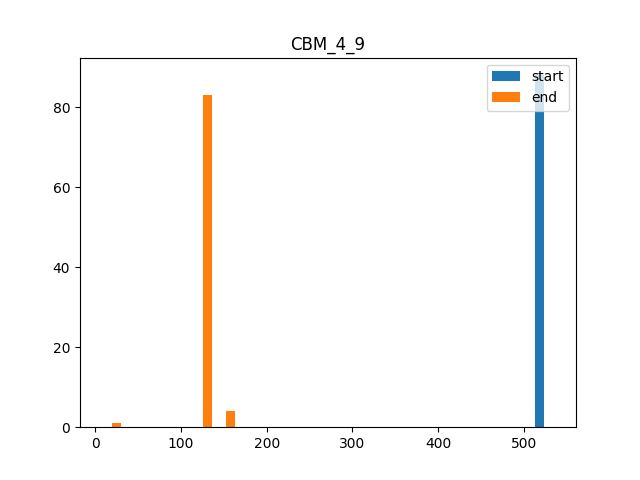

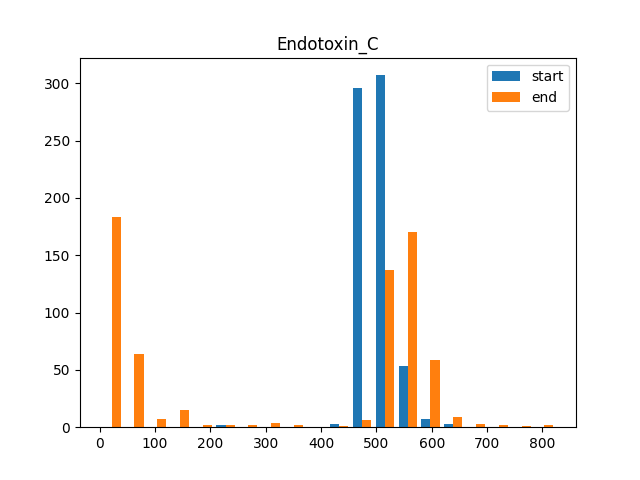


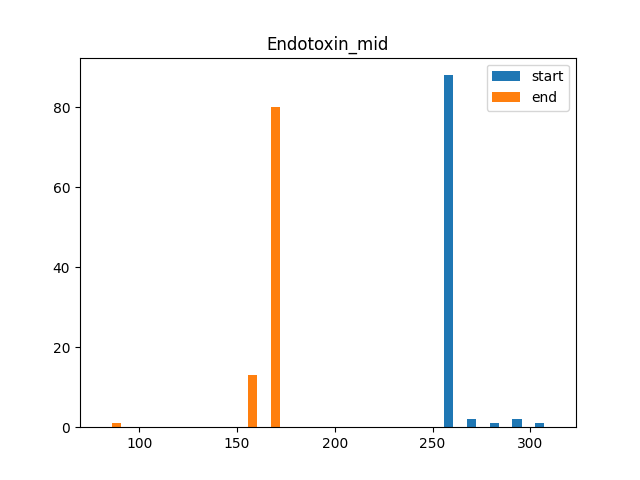

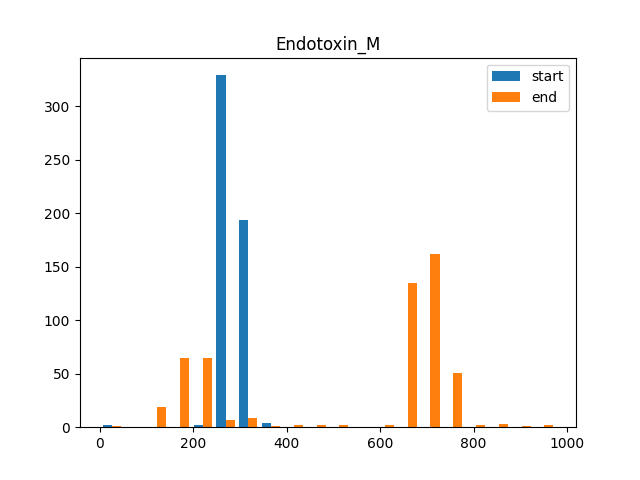


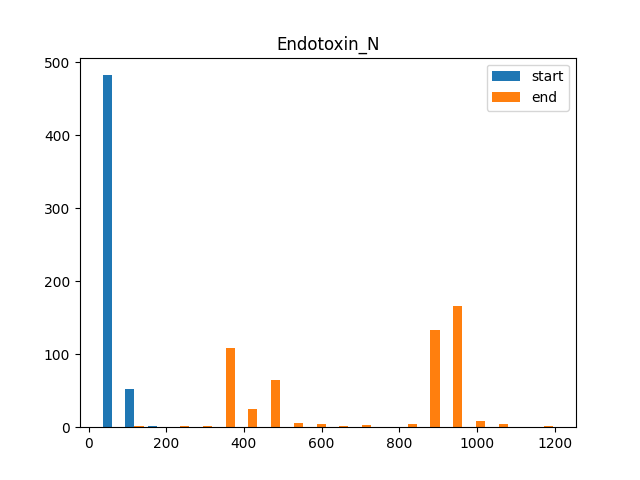

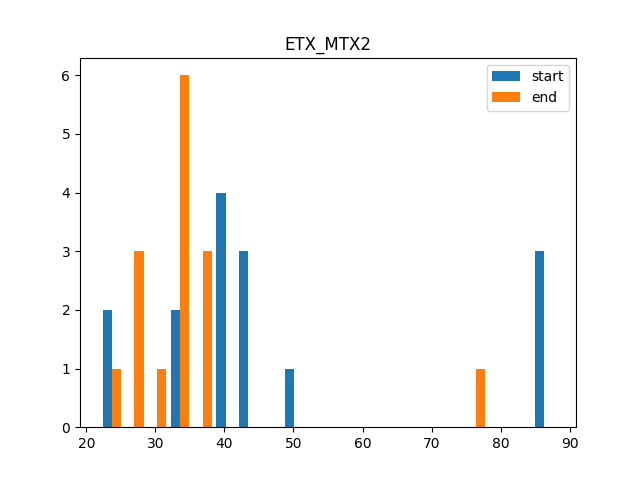


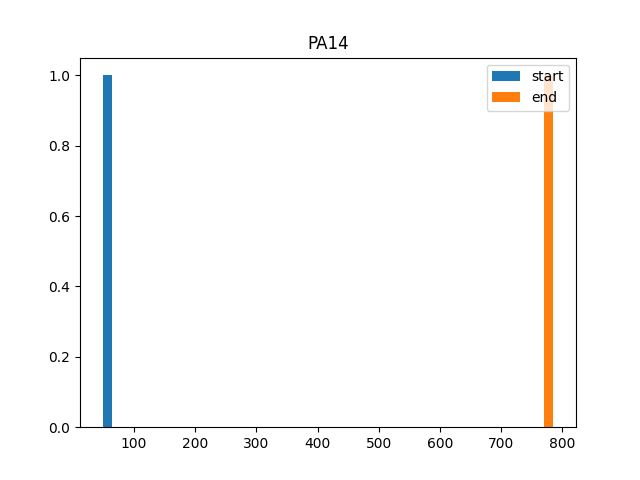

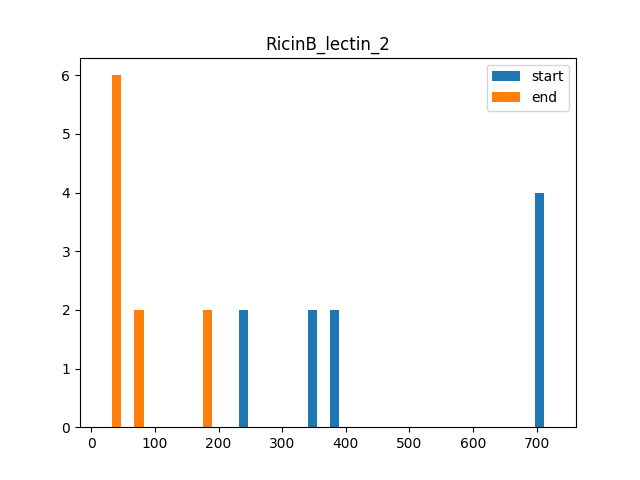


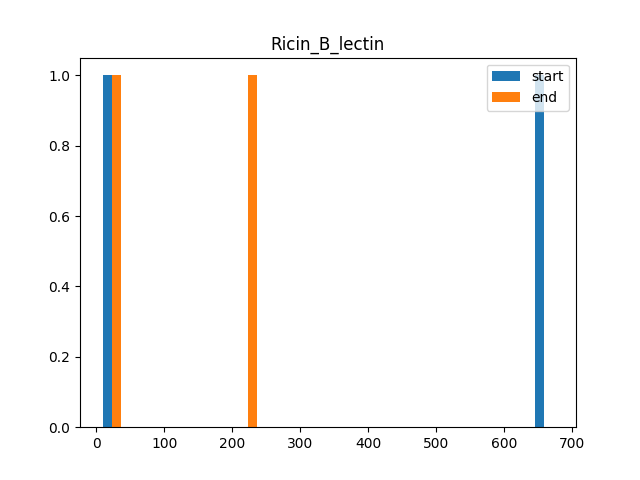

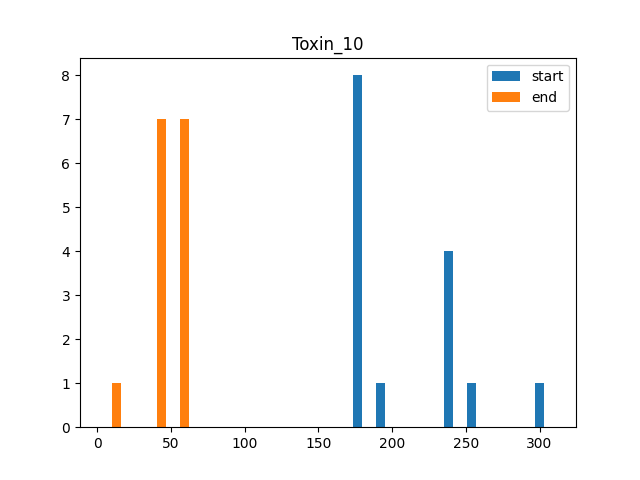


**Supplementary Figure S3. Histograms of locations of matches of HMMs along the IPG sequences (only 14 out of 66 HMM have reliable matches).** Orange (blue) columns show the distribution of distance from the gene starts (ends) to the location of the matches of HMMs.

# **Supplementary Note: Benchmarking ORFograph on simulated datasets**

**Datasets.** We analyzed one of the CAMI simulated dataset (Sczyrba, A. et al., 2017), complemented by simulated reads from various Bt strains that are enriched by the Cry genes. Specifically, we used the CAMI “TOY dataset” simulated from 30 genomes (<https://data.cami-challenge.org/participate>). The dataset contains 74 million paired-end reads of length 100 bp (mean insert size 180 bp). The errors in reads were modelled after Illumina HiSeq reads.

We took two complete assemblies of the Bt genomes from the NCBI database: BtA (NCBI ID=GCF_002025105) and BtB (NCBI ID=GCF_001598095) and aligned them to our IPG database formed with Cry, Cyt, and Vip proteins. We excluded plasmids with toxin-like sequences to have control over the Cry gene sequences that may be identified and to vary the complexity of the datasets. We excluded the plasmid NZ_CP020005.1 from BtA and the plasmids NZ_CP014852.1/NZ_CP014853.1 from BtB that contain partial or full alignments of toxins from the IPG database, forming the simulated datasets BtA_pure_ and BtB_pure_.

We varied the complexity of the IPG identification problem by enriching BtA_pure_ and BtB_pure_ with gradually increasing numbers of CDSs (0, 1, 2, 6) and with the gradually decreasing similarity between CDSs that varied from 45% to 92% (Tables S3 and S4).

| **Simulated Genome** | **Description** |
| --- | --- |
| BtA_pure_ | BtA without plasmid NZ_CP020005.1 |
| BtB_pure_ | BtB without plasmids NZ_CP014852.1, NZ_CP014853.1 |
| BtA_Cry1Aa1_ | BtA_pure_ with added Cry1Aa1 CDS in NZ_CP020003.1 at position 27668 |
| BtA_Cry1Aa1 + Cry1Ab1_ | BtA_Cry1Aa1_ with added Cry1Ab1 CDS in NZ_CP020004.1 atposition 24976 |
| BtA_Cry1Aa1 + Cry1Ac1_ | BtA_Cry1Aa1_ with added Cry1Ac1 CDS in NZ_CP020004.1 at position 12488 |
| BtA_Cry1Aa1 + Cry1Ab1 + Cry1Ac1_ | BtA_Cry1Aa1 + Cry1Ab1_ with added Cry1Ac1 CDS in NZ_CP020004.1 at position 12488 |
| BtB_Cry1Ca1 + Cry2Aa1 + Cry2Ac1_ | BtB_pure_ with added Cry1Ca1 CDS in NZ_CP020003.1 at  position 14716, Cry2Aa1 CDS in NZ_CP014848.1 at position 8614,  and Cry2Ac1 CDS at NZ_CP014849.1 position 19166 |

**Table S3. Information about seven simulated datasets enriched with Cry genes.**

| **NCBI ID** |  | cry1aa1 | cry1ab1 | cry1ac1 | cry1ca1 | cry2aa1 | cry2ab1 |
| --- | --- | --- | --- | --- | --- | --- | --- |
| **aaa22353** | cry1aa1 | 100 | 92 | 88 | 77 | 45 | 45 |
| **aaa22330** | cry1ab1 |  | 100 | 88 | 76 | 45 | 45 |
| **aaa22331** | cry1ac1 |  |  | 100 | 75 | 45 | 45 |
| **caa30396** | cry1ca1 |  |  |  | 100 | 45 | 46 |
| **aaa22335** | cry2aa1 |  |  |  |  | 100 | 89 |
| **aaa22342** | cry2ab1 |  |  |  |  |  | 100 |

**Table S4. Information about the percent identity between six analyzed Cry proteins.** Each cell shows the percent identity for between cry1aa1, cry1ab1, cry1ac1, cry1ca1, cry2aa1, and cry2ab1.

We simulated reads from Bt genome using the ART simulator (Huang et al., 2012) with “HS20 -l 100 -f 20 -m 180 -s 10” parameters (coverage 20x) and mixed them with reads from the CAMI “TOY dataset”. As a result we came up with five datasets described in Table S3.

**Results.** We launched ORFograph on the SPAdes (v14.1) assembly graphs and the contigs generated from the simulated read-sets listed in Table S3. To avoid false positive alignments of toxin HMMs that describe a wide range of toxins (rather than Cry proteins only), we used only the SPAligner alignments as anchors. Table S5 and Figures S4 and S5 illustrate ORFograph results.

We first tested whether ORFograph generates false positives by launching it on the NoCry dataset that contains no Cry gene. As expected, ORFograph did not identify any Cry genes in this dataset. In the case of a single Cry gene located on a single contig (dataset Cry1Aa1), ORFograph identified this Cry gene.

When we considered the Cry1Aa1+Cry1Ab1 dataset (with two similar Cry protein CDSs), SPAdes failed to assemble them. ORFograph, on the other hand, reconstructed both these genes, as well as generated two chimeric sequences (Figure S4). In the case of the Cry1Aa1+Cry1Ac1 dataset (with two distant Cry genes with similarity only 88%), ORFograph has separated Cry1Aa1 and Cry1Ac1 CDSs and identified a substring of Cry1Aa1 as ORF.

When we considered all six Cry CDS present in the metagenome, ORFograph identified four clusters of ORFs: three of them represent Cry1-like proteins’ CDS and one represent Cry2-like proteins’ CDS. Figure S5 shows a complex subgraph, where paths of Cry1-like proteins’ CDS are located.

Our analysis of the simulated dataset demonstrated that ORFograph successfully identifies Bt genes but may “glue” very similar sequences. It may also generate spurious genes when (i) there is limited information available from contigs or (ii) due to imperfect representative selection procedure.

| **#** | **Dataset Name** | **TOY dataset+**  **simulated reads** **from:** | **Unassembled by SPAdes** | **ORFograph results** |
| --- | --- | --- | --- | --- |
| **1** | NoCry | BtA_pure_ and BtB_pure_ | - | No Cry genes identified |
| **2** | Cry1Aa1 | BtA_Cry1Aa1_ | - | One cry1a-like CDS |
| **3** | Cry1Aa1 + Cry1Ab1 | BtA_Cry1Aa1 + Cry1Ab1_ | cry1aa1, cry1ab1 | Four sequences similar to cry1aa1 and cry1ab1 CDS |
| **4** | Cry1Aa1 + Cry1Ac1 | BtA_Cry1Aa1 + Cry1Ac1_ | cry1aa1, cry1ac1 | Three sequences: one is identical to cry1ac1 CDS, and the other two are similar to cry1aa1 |
| **5** | AllCry | BtA_Cry1Aa1 + Cry1Ab1 + Cry1Ac1_  and  BtB_Cry1Ca1 + Cry2Aa1 + Cry2Ac1_ | cry1aa1, cry1ab1,  cry1ac1, cry1ca1 | Four clusters of ORFs: one Cry2-like (which belongs to one contig consisting of three edges) and three clusters of Cry1-like proteins: the first cluster contains cry1aa1 and cry1ab1-like sequences, the second cluster contains cry1aa1 and cry1ac1-like sequence, and the third cluster represents the cry1ca sequence |

**Table S5. Benchmarking ORFograph on five simulated datasets of varying complexity.**


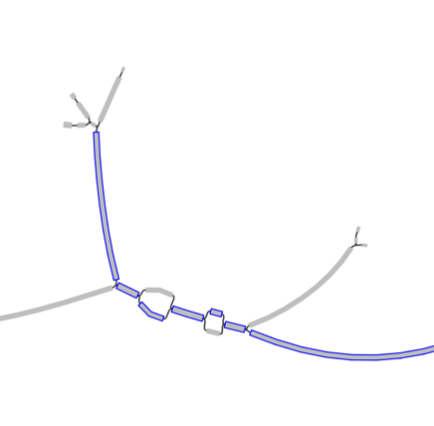

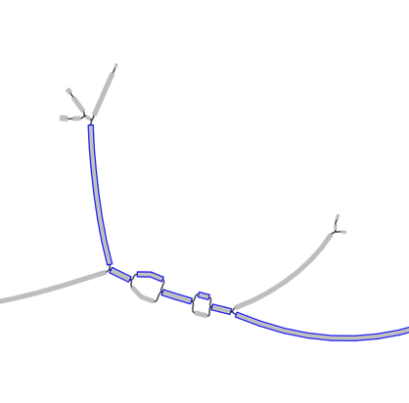

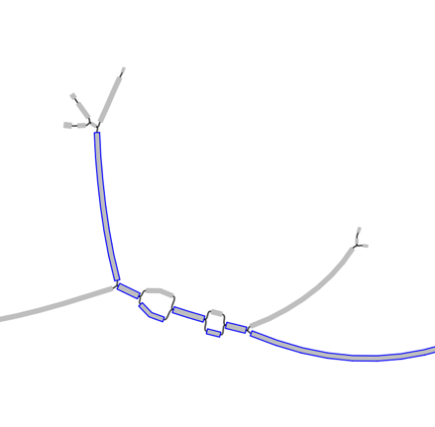


**Figure S4. Most reliable IPGs (paths) identified by ORFograph in the Cry1Aa1+Cry1Ab1 dataset.** Grey edges represent the subgraph containing all paths, while blue edges represent paths of four potential IPGs identified by ORFograph. In this subgraph, each edge represents a separate contig, illustrating that ORFograph faced the challenge of filtering spurious paths in the absence of the contig-related information from SPAdes. The shortest edge in the shown subgraph has length 348 bp.


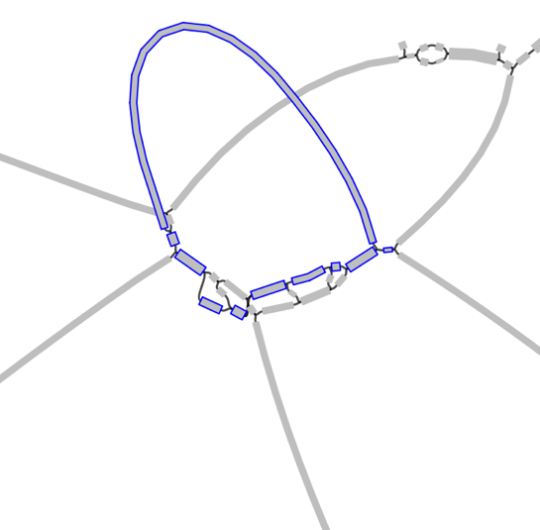


**Figure S5. The subgraph (grey edges) and the path (blue edges) of the Cry1-like IPG generated by ORFograph for the AllCry dataset.**

# **Supplementary Note: Benchmarking ORFograph against plasmidSPAdes and metaplasmidSPAdes.**

**Analyzing plasmidSPAdes and metaplasmidSPAdes results for B_ALL_ and NYCS datasets.** We launched plasmidSPAdes (Antipov et al., 2016) on 40 B_ALL_ datasets where ORFograph found 232 novel IPGs scattered across several contigs.

In order to identify which of 232 novel IPGs are encoded in plasmids (assembled by plasmidSPAdes), we aligned the plasmidSPAdes contigs against a set of 232 novel IPGs using the Diamond aligner (Buncfink et al., 2015) and classified alignments with identity exceeding 95% and length exceeding 95% of the IPG length as *plasmidic*. This analysis revealed that only 34 out of 232 IPGs were classified as plasmidic. These 34 sequences belong to 9 out of 40 analyzed datases (ERR498188, ERR498164, SRR9189225, SRR6238356, ERR498200, SRR642765, SRR8846359, SRR6238358, ERR498165, SRR634070, SRR6238345, SRR9189593).

Afterward, we aligned plasmidSPAdes contigs to the SPAdes assembly graph using SPAligner (in the nucleotide mode) to analyze the relation between the IPG-paths and the plasmid-paths in the SPAdes assembly graph. For 180 out of 232 IPGs, at least one edge from the IPG-path was missing in plasmid-paths (we removed the first and the last edge of each IPG-path from consideration).

This analysis revealed the advantages of ORFograph over plasmidSPAdes. The limited power of plasmidSPAdes in IPG identification is explained by the fact that plasmidSPAdes filters out an edge from the SPAdes assembly graph if its coverage is similar to the median coverage (or if it represents a dead-end edge). Thus, if a plasmid-edge has coverage similar to the median coverage, plasmidSPAdes classifies it as non-plasmidic and filters it out. Our analysis revealed that it is often the case. Thus, using plasmidSPAdes (as compared to SPAdes) has limited benefits for the IPG identification, particularly with respect to identifying Cry genes that are typically located on long plasmids.

We also launched metaplasmidSPAdes (Antipov et al., 2019) in order to compare the set of Cry genes identified by ORFograph and the set of Cry genes identified in the metaplasmidSPAdes contigs in the NYCS dataset. The Diamond tool did not find any alignments of the Cry genes to contigs generated by metaplasmidSPAdes. This finding illustrates that using metaplasmidSPAdes (as compared to SPAdes) has limited benefits for the IPG identification.

**References for Supplementary Notes**

Buchfink, B., Xie, C., & Huson, D. H. (2015). Fast and sensitive protein alignment using DIAMOND. Nature methods, 12(1), 59–60. <https://doi.org/10.1038/nmeth.3176>

Huang, W., Li, L., Myers, J. R., & Marth, G. T. (2012). ART: a next-generation sequencing read simulator. Bioinformatics, 28(4), 593–594. https://doi.org/10.1093/bioinformatics/btr708

###

### 
